# Parameter Identifiability and Sensitivity Analysis Predict Targets for Enhancement of STAT1 Activity in Pancreatic Cancer and Stellate Cells

**DOI:** 10.1371/journal.pcbi.1002815

**Published:** 2012-12-20

**Authors:** Katja Rateitschak, Felix Winter, Falko Lange, Robert Jaster, Olaf Wolkenhauer

**Affiliations:** 1 Department of Systems Biology and Bioinformatics, University of Rostock, Rostock, Germany; 2 Department of Medicine II, Division of Gastroenterology, University Medicine Rostock, Rostock, Germany; 3 Stellenbosch Institute for Advanced Study (STIAS), Wallenberg Research Center at Stellenbosch University, Stellenbosch, South Africa; Accelrys, United States of America

## Abstract

The present work exemplifies how parameter identifiability analysis can be used to gain insights into differences in experimental systems and how uncertainty in parameter estimates can be handled. The case study, presented here, investigates interferon-gamma (IFNγ) induced STAT1 signalling in two cell types that play a key role in pancreatic cancer development: pancreatic stellate and cancer cells. IFNγ inhibits the growth for both types of cells and may be prototypic of agents that simultaneously hit cancer and stroma cells. We combined time-course experiments with mathematical modelling to focus on the common situation in which variations between profiles of experimental time series, from different cell types, are observed. To understand how biochemical reactions are causing the observed variations, we performed a parameter identifiability analysis. We successfully identified reactions that differ in pancreatic stellate cells and cancer cells, by comparing confidence intervals of parameter value estimates and the variability of model trajectories. Our analysis shows that useful information can also be obtained from nonidentifiable parameters. For the prediction of potential therapeutic targets we studied the consequences of uncertainty in the values of identifiable and nonidentifiable parameters. Interestingly, the sensitivity of model variables is robust against parameter variations and against differences between IFNγ induced STAT1 signalling in pancreatic stellate and cancer cells. This provides the basis for a prediction of therapeutic targets that are valid for both cell types.

## Author Summary

For the prediction of therapeutic targets and the design of therapies, it is important to study the same pathway across different cell types. This is particularly relevant for cancer research, where several cell types are involved in carcinogenesis. Pancreatic cancer is enhanced by activated pancreatic stellate cells. It would thus seem plausible for an effective therapy to hit stellate and cancer cells. The cytokine IFNγ is an inhibitor of proliferation in both cell types. Antiproliferative effects of IFNγ are mediated by STAT1 signalling. An important aspect is to determine those reactions that cause the differences in the initial increase of phosphorylated STAT1 and in the temporal profile of STAT1 nuclear accumulation between the two cell types. We examined this aspect by performing a parameter identifiability analysis for calibrated mathematical models. We calculated confidence intervals of the estimated parameter values and found that they provide insights into reactions underlying the differences. A key finding of sensitivity analysis elucidated that predicted targets for enhancement of STAT1 activity are robust against parameter uncertainty and moreover they are robust between the two cell types. Our case study therefore exemplified how identifiability and sensitivity analysis can provide a basis for the prediction of potential therapeutic targets.

## Introduction

Progression of pancreatic cancer (PC) is accelerated by an extended fibrosis, which has been linked to the activation of pancreatic stellate cells (PSC) [1]–[3]. Therefore therapies will be particularly effective if they simultaneously hit carcinoma and stroma cells. IFNγ acts as an antagonist of PSC activation and displays inhibitory effects on PC growth by inducing the STAT1 signalling pathway in both cell types [4], [5]. While the qualitative effects of IFNγ were the same in cancer and stellate cells, a quantitative analysis revealed significant differences. Specifically, IFNγ inhibited PSC proliferation more efficiently than tumour cell growth. The stronger biological effect of IFNγ in PSC correlated with a more pronounced nuclear accumulation of STAT1 in the stroma cells [4]–[6], raising the question which molecular mechanisms are underlying these observations.

IFNγ-induced STAT1 signalling was investigated by combining experimental and theoretical systems biology in [5], [6]. The same structure of an ordinary differential equation (ODE) model could be used for both cell types. The parameter values were estimated from experimental time series for STAT1 phosphorylation and protein expression and expression of the STAT1 target gene suppressor of cytokine signaling 1 (*SOCS1*) using global optimization.

The present work is motivated by the following consideration: We observed differences in the trajectories of the parameterized models describing the IFNγ-induced STAT1 signalling pathway in PSC and cancer cells. Simulation results agreed with the differences in the experimental time series. The differences can thus be attributed to unequal parameter values of the two models. In general, however, one finds a range of parameter sets leading to a similarly good fit of the model, rather than a unique parameter set. As a consequence it remains unclear which specific model parameters are behind the differences observed for the model trajectories of the two cell types. We therefore focused on the following two questions:

1. *Which reactions cause the differences in the temporal profiles of the model simulations between PSC and cancer cells?*
2. *What are the consequences for therapeutic target prediction?*

An appropriate method for our first objective is a parameter identifiability analysis. It investigates how accurate the parameter values are determined by the model structure including a mapping of model variables to observables [7], [8] and by the experimental data [8]. This analysis is necessary because successful parameter estimation usually results in a range of parameter sets that realise an equally good agreement between experimental data and model simulations. In this work, we adopted the approach of [8], which is based on calculating a confidence interval (CI) for each parameter value. Consequences of uncertainties in parameter values can be studied by comparing model trajectories resulting from parameter sets within the CI [9]. This enabled us to identify those reactions that are underlying the differences in the model simulations between both cell types. We therefore exploited the results of parameter identifiability analysis for a systematic parameter comparison of two structurally identical mathematical models, which is - to our best knowledge - the first time such an analysis was conducted.

Note that our approach here is different to typical methods of model discrimination used in systems biology that compare for two models with a different number of parameters the quality of the fits to the same experimental data. These methods investigate whether a model with more parameters than a default model describes given data better, without introducing more uncertainty in the parameter values. Such approaches include Akaike Information Criterion [10], [11], likelihood ratio test for nested models [10]–[12], Bayesian model comparison [13] and stimulus design for model selection [14].

In the next part of this work, we investigated the consequences of parameter uncertainties for the prediction of common therapeutic targets in both cell types. We performed a sensitivity analysis to determine those parameters, which sensitively influence protein concentrations during the time of observation. As a quantitative measure for sensitivity we employed what is known as the “metabolic control coefficient” [15], [16]. We used our results from parameter identifiability analysis to select parameter sets for sensitivity analysis. Sensitive parameters are discussed as candidates for therapeutic targets in both cell types.

It is worth pointing out that we confirmed the key findings of the study in a larger panel of pancreatic cancer cell lines and in primary PSC. Finally, we discussed our results from experiments, parameter identifiability analysis and sensitivity analysis in the light of STAT1 nuclear accumulation.

## Methods

### Estimation of parameter values

The parameters of the two models include reaction constants, delay times, total receptor concentration, Western blot scaling factors and initial conditions of some model variables. Their values were estimated by global optimization from protein and mRNA time series data. We used the experimental findings of reference [5], [6] and additionally raised data of phosphorylated STAT1 translocation in PSC. The additional time series contributed to stable profile likelihood estimates presented in the “Results” section.

For parameter value estimation a hybrid approach was applied, which is a combination of a stochastic simulated annealing algorithm, performing a global search, and a deterministic trust region algorithm performing a local search. The hybrid approach is implemented in the routine pwFitBoost of the MATLAB toolbox PottersWheel [17]. As a measure for how good a simulation of the model reproduces experimental data, the following cost function was used:
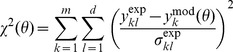
(1)where 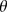 is the parameter vector, 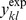 are the experimental data, 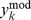 are values of observables at time points when experimental data are measured, 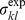 is the measurement error of the experimental data, 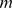 is the number of time points and 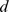 is the number of observables [17]. We repeated the parameter value estimation 50 times and varied the start set of values to ensure that we approach the global minimum of 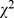 as close as possible. The parameter value set for the PSC model and the parameter value set for the PC model presented in Tables S1, S2, S3 in Text S1 belong to the best fitting sets. For PSC it is the set which leads to the PLE minimum of 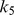 and 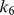 in section “Results”. For PC it is the set with 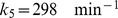 which is located within the plateau of the PLE in section “Results”.

Due to nonlinearities in the models averages of parameter values do not necessarily lead to a good fit and are thus not appropriate quantities for further analysis. Instead of this, a parameter identifiability analysis provides a confidence interval with a confidence level for each parameter value.

### Parameter identifiability analysis

A parameter identifiability analysis answers the question of how accurate the parameter values of a given model can be determined by the experimental data. This in turn allows an investigation of which model predictions are possible [9]. We apply the data-based approach by Raue et al. [8], which is based on calculating CIs defined by a threshold in the profile likelihoods. Following the authors: “A parameter is identifiable, if the confidence interval of its estimate is finite.” The approach is summarized below.

The value 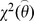 of Eq. (1) corresponds to the maximum likelihood estimate for Gaussian measurement noise, where 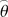 denotes the parameter set of the best fit. Thus we can use 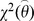 as a related expression for the maximum likelihood estimate. Profile likelihood estimates (PLE) are calculated by iteratively shifting one parameter from its optimal value by a small value followed by fitting again the other parameters [8], [18]. This procedure continues either until a threshold in the likelihood is met or until a maximal number of iterations have passed. Based on a likelihood threshold, a CI for a parameter estimate can be defined [8], [19]:
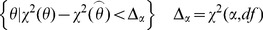
(2)


The degrees of freedom (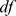) give for 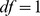 the point-wise and for 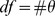 the simultaneous CI (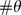 denotes number of parameters), both to the confidence level α. We used the simultaneous CI to assure that the majority of parameters are within the CI when simulating trajectories. Changing a parameter value within its CI the model observables stay in agreement with the experimental noise.

Structural nonidentifiability is caused by a redundant parameterisation of the mathematical model. Such correlated parameters lead to a curve with the same likelihood value in the high dimensional landscape of the likelihood function. The CIs are infinite. Practically nonidentifiability arises if the amount of experimental data is insufficient with respect to the complexity of the mathematical model. In that case 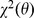 increases by moving away from the best fit but the CI of the parameter value is infinite at least in one direction. The 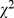
- curve of a practical nonidentifiable parameter can be very flat with a minimum difficult to detect as in our study. The approach of [8] is in contrast to other published methods, which only allow uncovering structural nonidentifiability and could be more difficult to apply for larger nonlinear models [7].

The difference 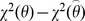 is equal to the amount of overfitting by the parameter set 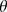 [20]. To test whether a model shows deviations from the 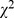 distribution, random time series are generated with the same mean and the same standard deviation as the original data [20], [21]. The model is then successively fitted against all random data sets. Next the optimized parameter values for each random data set are used to calculate 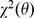 respective to the original data set according to Eq. (1). Finally, the distribution of 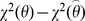 is compared with a 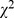 distribution with 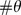 degrees of freedom. Deviations from a distribution with 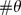 degrees of freedom indicate that the degrees of freedom of the model are lower than the number of parameters.

### Sensitivity analysis

A sensitivity analysis answers the question how small perturbations of a single or multiple parameter values influence the trajectories of arbitrary model variables [22]. For this analysis, it is irrelevant whether the model parameter values are arbitrarily chosen or the result from fitting the model to experimental data.

We are interested in the time-dependent sensitivity of a specific model variable, as a function of individual model parameter perturbations. A simple appropriate measure is the metabolic control coefficient. They measure the relative response of a state variable 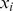 with respect to the relative perturbation by the parameter 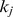 and are a standard quantitative measure in sensitivity analysis [15]. Metabolic control coefficients can be also calculated for finite times as described in [16]. They are defined by
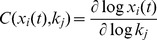
(3)


If 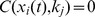 then the parameter perturbation has no influence on the model variable at the respective time point. The larger the value of 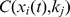 the higher is the influence of the parameter perturbation. For each parameter the sensitivity analysis was performed for 20 parameter sets covering the CI. In each of the 20 sets the value of the default parameter was perturbed by −1% to calculate the metabolic control coefficient. This procedure allows to study whether nonidentifiability influences the results of sensitivity analysis.

### Complementary experiments

#### Cell culture

Immortalised rat PSC [23] were cultured in Iscove's modified Dulbecco's medium (IMDM; Biochrom, Berlin, Germany) supplemented with 17% fetal calf serum (FCS) (PAA Laboratories, Pasching, Austria), 1% non-essential amino acids, 100 U/ml penicillin and 100 µg/ml streptomycin. Primary rat PSC were isolated as described before [24] and cultured in IMDM supplemented with 17% FCS, 1% non-essential amino acids, 100 U/ml penicillin and 100 µg/ml streptomycin. The PC cell lines BxPC-3, MIA PaCa-2 and DSL-6A/C1 were obtained from the American Type Culture Collection (ATCC). BxPC-3 was cultured in RPMI 1640 medium supplemented with 10% FCS, 100 U/ml penicillin and 100 µg/ml streptomycin. MIA PaCa-2 and DSL-6A/C1 were cultured in Dulbecco's modified Eagle medium supplemented with 10% FCS, 100 U/ml penicillin and 100 µg/ml streptomycin. The cells were grown at 37°C in a 5% CO_2_ humidified atmosphere.

#### Immunofluorescence staining

All cell types were cultured onto glass coverslips until reaching approximately 20% confluence. The cells were stimulated with 100 ng/ml species-specific IFNγ (Immunotools, Friesoythe, Germany) for up to 9 hours and fixed in methanol. Immunofluorescence staining of cytoplasmic and nuclear STAT1 and phospho-STAT1 as well as confocal laser scanning microscopy were done as described in [5], [6]. Briefly, images were further analysed using the software ImageJ (Open Source software package). To quantify nuclear translocation of STAT1, averaged ratios of nuclear versus cytoplasmic concentration (sum of pixel intensity divided by area) of STAT1 were calculated. Therefore, at least 10 cells from three different images were analysed for each time point.

## Results

### Experimental data and model simulations reveal differences between pancreatic stellate and cancer cells

**Figure 1:**
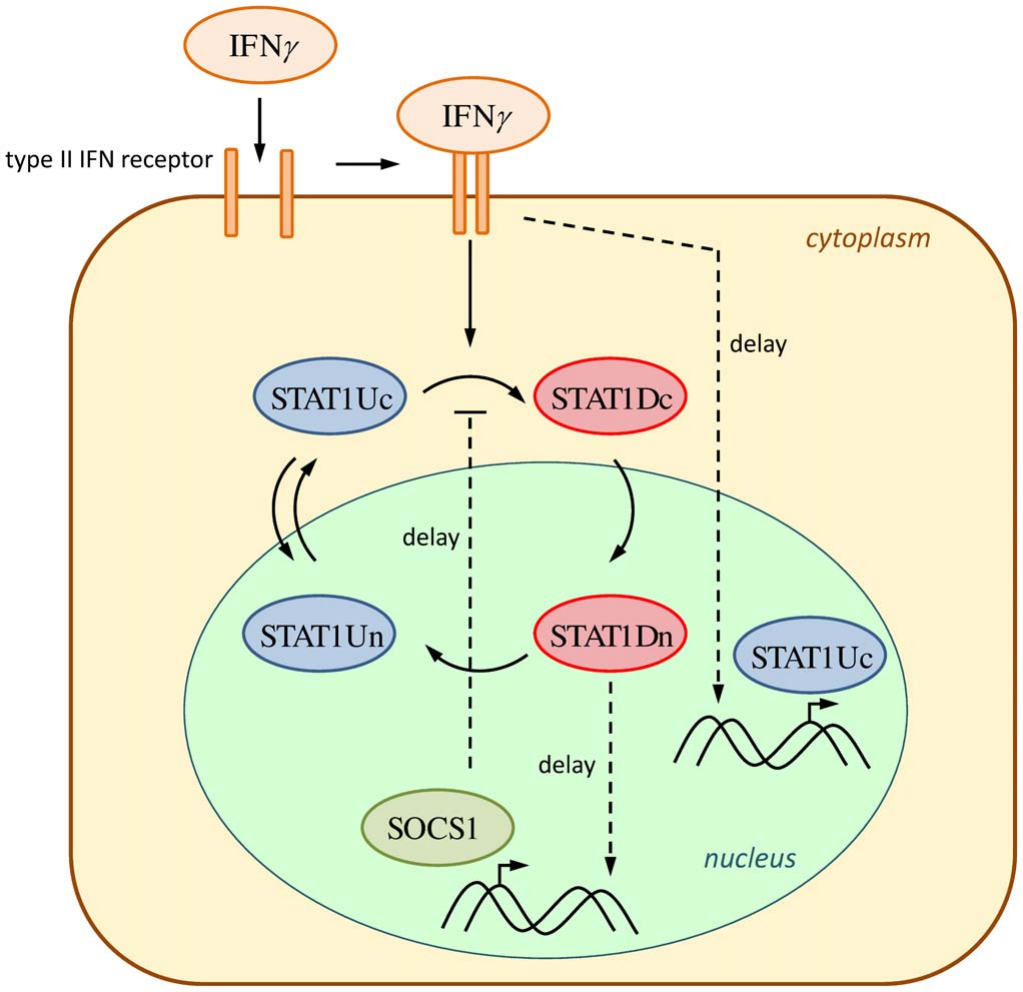
Reaction network of the IFNγ stimulated STAT1 signalling pathway. The network shows key reactions of the pathway. IFNγ activates the type II IFN receptor. To keep the model simple, Janus kinases (JAK) are not considered separately but as part of the active receptor complex only. The receptor-associated JAKs phosphorylate cytosolic STAT1 (STAT1Uc), followed by rapid and high affinity formation of homodimers (STAT1Dc). STAT1Dc translocates into the nucleus (STAT1Dn). Nuclear STAT1D can be dephosphorylated, leading to nuclear export of the resulting STAT1Un into the cytoplasm. STAT1Uc can also shuttle into the nucleus. As a transcription factor, STAT1 induces the transcription of specific target genes. The network considers *STAT1* itself and *SOCS1* as target genes of IFNγ-activated signalling. *SOCS1* is a potential negative feedback regulator; inhibiting the phosphorylation of STAT1Uc. The annotation *delay* refers to temporal differences between IFNγ action at the receptor level and consecutive steps. We could omit the binding of nuclear phosphorylated STAT1 to the DNA in this work comparing to [5], [6], because the slightly simplified model leads to indistinguishable fits, as shown in Figures S1 and S2 in Text S1.

**Figure 2:**
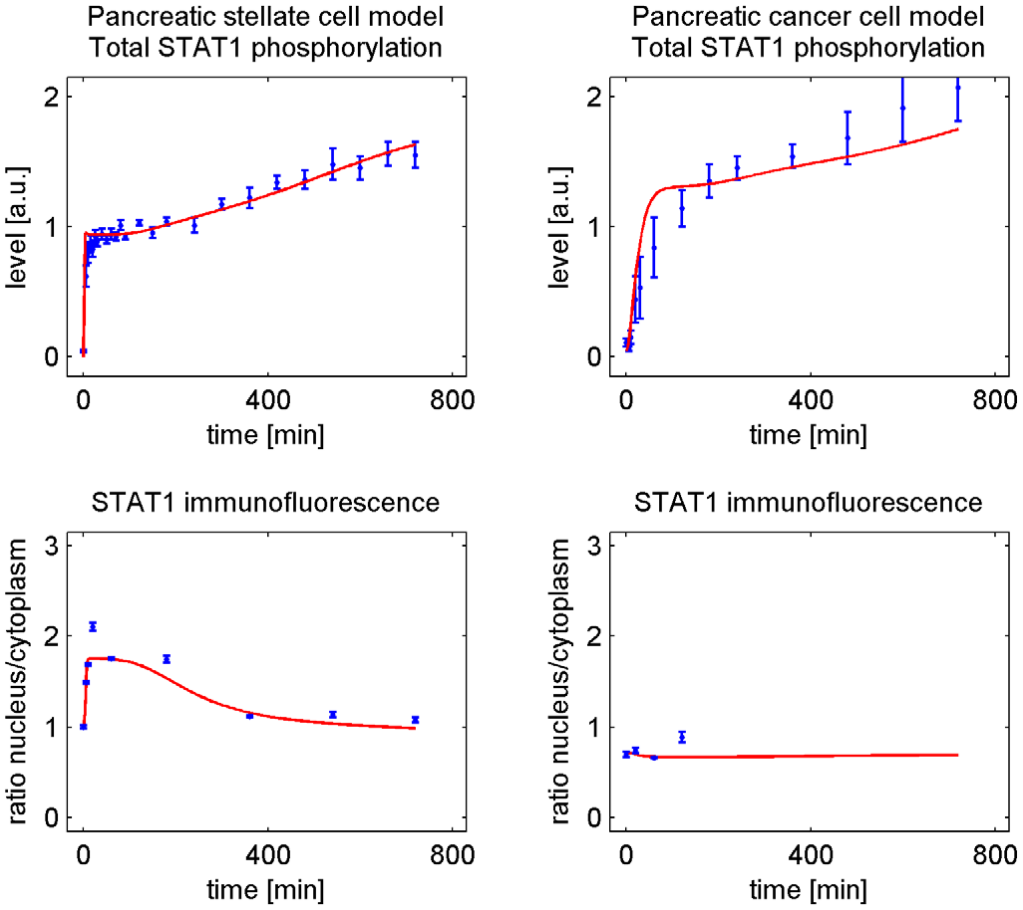
IFNγ-induced STAT1 pathway in PSC and PC: Comparison between experimental time series and model simulations. Experimental time series and model simulations that differ between the two cell types are shown. The left column in Figure 2 contains two subfigures of Figure S1 in Text S1 and the right column in Figure 2 contains two subfigures of Figure S2 in Text S1 for IFNγ = 100 ng/ml. The observation time is given on the x-axis of each subfigure. Experimentally determined expression levels of phospho-STAT1 protein are given in arbitrary units (a.u.). Immunofluorescence analysis data obtained by confocal microscopy were processed by calculating the ratio of nuclear versus cytoplasmic STAT1 concentration. Measured data are presented as blue circles with error bars. The simulated time courses resulting from the mathematical model with optimized parameter values are presented by red solid lines. The quality of the fit in the upper right subfigure is commented in the captions of Figure S2 in Text S1. Experimental time series are replotted from [5], [6].

The network presented in Figure 1 shows the key reactions of the IFNγ-induced STAT1 pathway (cf. [5], [6]). The network was translated into a system of ODEs, which is presented in the supporting information. The simulations of the parameterized ODE models are in good agreement with the experimental time series for both cell types (Figures S1 and S2 in Text S1). Interestingly, the experimental time series and the model simulations revealed two major differences between PSC and PC summarized in Figure 2:

- Rapid initial STAT1 phosphorylation in PSC. Slower initial rise of phosphorylated STAT1 in PC.
- Nuclear accumulation of STAT1 in PSC. Lack of nuclear accumulation of STAT1 in PC.

This raised the question which reactions caused these differences in the model simulations between PSC and PC. To approach this question we performed a parameter identifiability analysis.

### Parameter identifiability analysis

**Figure 3:**
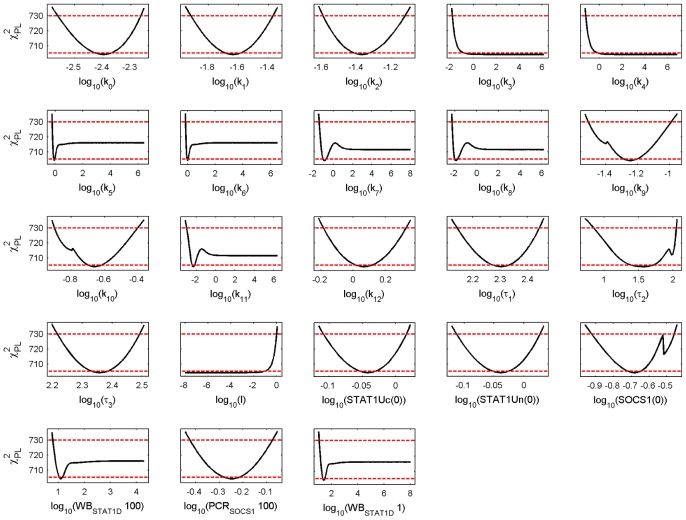
Profile likelihood estimates for the calibrated PSC model. Model parameters or initial conditions of variables are given on the x-axis of each subfigure. The PLE (black line) together with the point wise (red dashed lower horizontal line) and simultaneous confidence levels (red dashed upper horizontal line) are shown on the y-axis. The values of the x-axis where the PLE crosses the confidence levels yield the lower and upper boundary of the point wise and simultaneous confidence intervals, respectively. A parameter is identifiable if both confidence intervals are finite. We used the simultaneous confidence levels.

**Figure 4:**
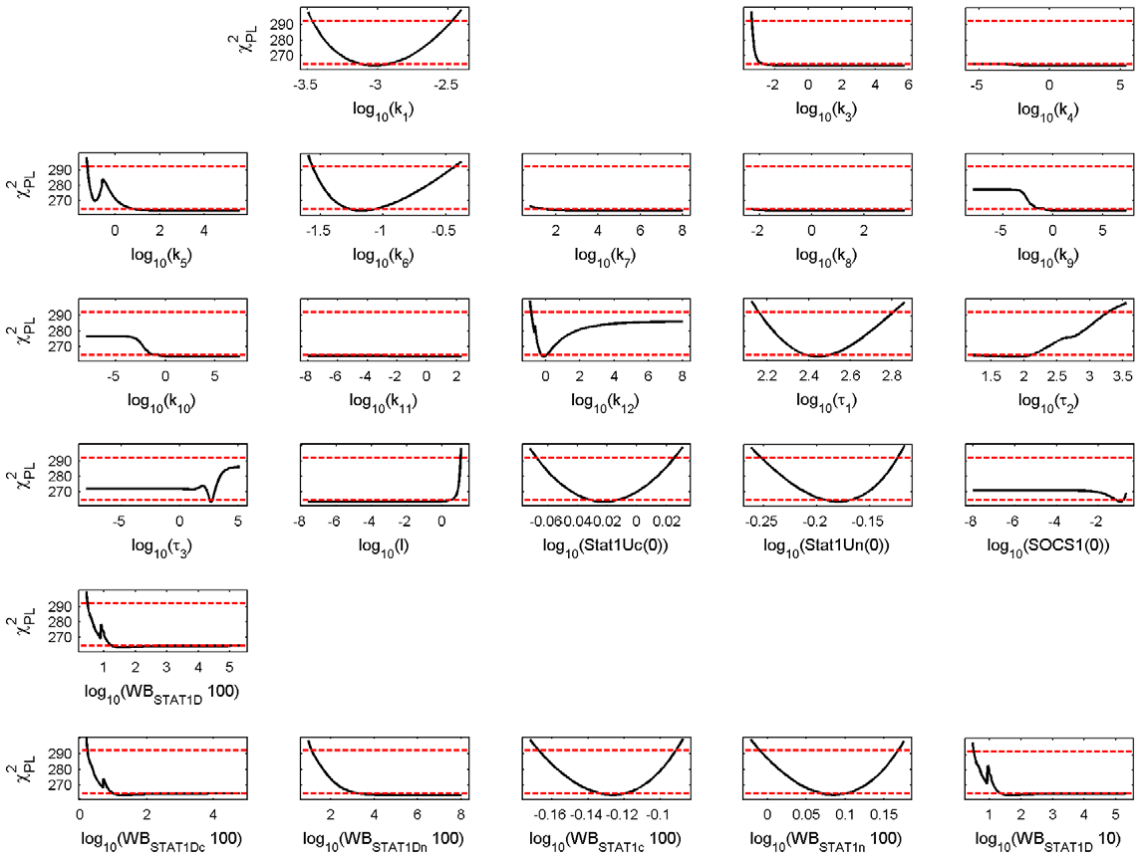
Profile likelihood estimates for the calibrated PC model. Model parameters or initial conditions of variables are given on the x-axis of each subfigure. The PLE (black line) together with the point wise (red dashed lower horizontal line) and simultaneous confidence interval (red dashed upper horizontal line) are shown on the y-axis. For further details see Figure 3.

We performed a parameter identifiability analysis by calculating the profile likelihood estimates (PLE). The PLE for the parameter values of the PSC model are shown in Figure 3 and the PLE for the parameter values of the PC model are shown in Figure 4. The PLE profiles of both cell types show identifiable and nonidentifiable parameters.

A distinction between the PSC and PC profiles, important for our later discussion, is parameter 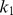 that describes the receptor activation and parameter 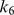 that describes nuclear import of phosphorylated STAT1. The lower boundary of the CI for the PSC value and the upper boundary of the CI for the PC value do not overlap and the parameter value at the minimum is for both 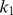 and 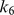 more than ten-fold higher for the PSC model than for the PC model. In contrast, the parameter of feedback inhibition (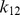) has the same minimal value for the PSC and PC model but the upper CI is marginally missed by the PLE for PC. The parameter describing STAT1 phosphorylation (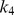) has a finite lower CI boundary at around zero for PSC whereas is in unbounded for PC. The parameter for nuclear export (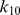) is identifiable for PSC but without CI boundaries for PC.

#### Deviation from 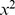 distribution

**Figure 5:**
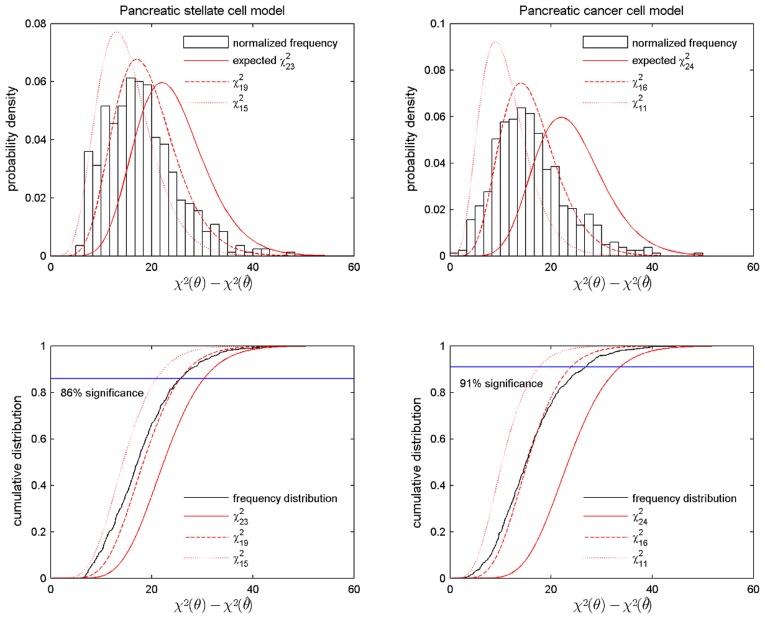
Amount of overfitting of the calibrated PSC and PC model. Upper figures: Comparison of the normalized frequency distribution 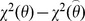 with the probability distribution 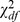. Lower figures: Respective cumulative distributions. The 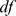 are chosen to represent the theoretically expected degrees of freedom (solid line), the degrees of freedom most compatible with the frequency distribution (dashed line) and the calculated degrees of freedom (dotted line). Left column: results for the calibrated PSC model, right column: results for the calibrated PC model.

According to Eq. (2) we calculated the amount of overfitting for the two models. The comparison between the normalized frequency distribution of 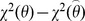 and the 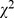 probability distributions with different degrees of freedom is shown in the upper row of Figure 5 for both models. We observed deviations between the calculated normalized frequency distribution and the expected 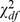 with 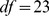 for the PSC and 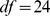 for the PC model (solid lines). These deviations could be caused by the nonlinearities in the model and by the small data sets [20], [21]. Reducing the value of 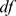 by the number of large-range correlated parameters leads to 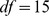 for the PSC model 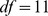 for the PC model. However, we still observe deviations between the calculated 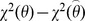 and the expected 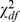 (dotted lines) for both models. Therefore we finally chose intermediate values as “effective number of degrees of freedom” of 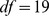 for the PSC model and 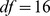 for the PC model. Simulations show that the 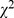 curve (dashed lines in upper row of Figure 5) and the histogram over 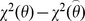 agree very well.

We furthermore plotted the cumulative distribution function in the lower part of Figure 5, which allows either calculating appropriate confidence levels for the given confidence intervals or alternatively appropriate confidence intervals for the given confidence levels for the estimated parameter values. The calculated CI led to a higher confidence level 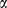 of 86% for the PSC model and of 91% for the PC model. For the initial assumption that the degrees of freedom are equal to the number of parameters the confidence level was 68%. Alternatively we could have used the expected confidence intervals with the original confidence levels, which would have not changed our results of the identifiability analysis.

#### Reactions which cause differences between the temporal profiles of PSC and PC

Consequences of uncertainties in parameter values on the dynamics can be studied by comparing trajectories resulting from parameter sets within the confidence interval. In the following, we focus on parameters, which based on their position in the biochemical network, could be related to the different experimental profiles of STAT1 and phosphorylated STAT1 between PSC and PC after stimulation with IFNγ.

**Figure 6:**
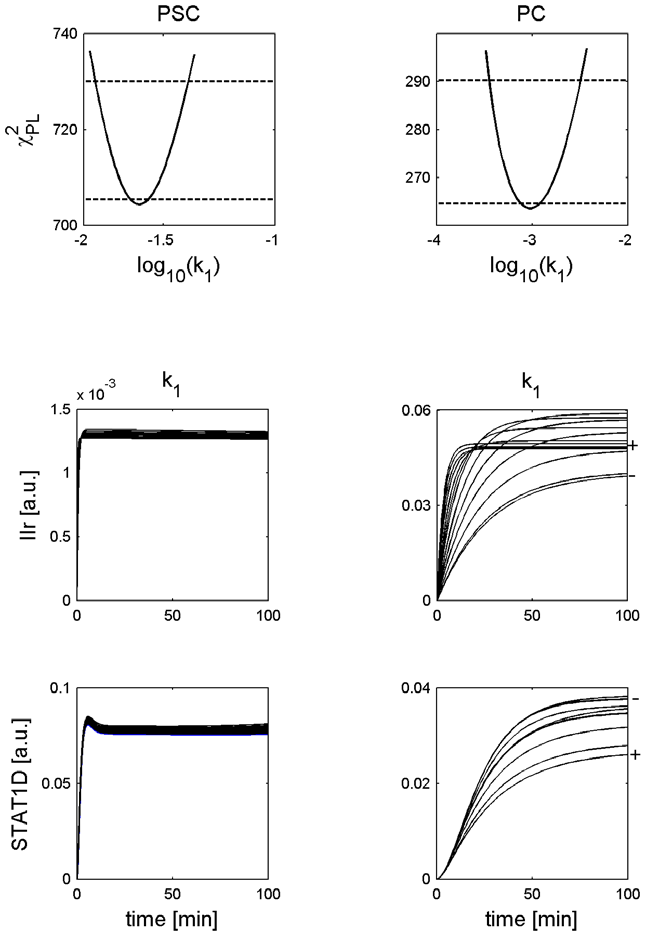
Trajectories for parameter sets along profile likelihoods show differences in phosphorylated STAT1 increase. The upper row shows the PLEs of the parameter 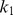 from Figures 3 and 4. The lower rows show trajectories generated from 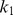-parameter sets within the PLE confidence intervals of the variables active receptor concentration (IIr) and phosphorylated STAT1 (STAT1D) for both cell types stimulated with IFNγ = 100 ng/ml. The observation time is given on the x-axis of each subfigure. The trajectories for 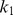-parameter sets are shown on the y-axis. For good visibility approximately 11 trajectories are plotted in equal distance according to the PLE in each subfigure. A “−” indicates the location of the trajectory for the smallest parameter value. A “+” indicates the location of the trajectory for the largest parameter value.

**Figure 7:**
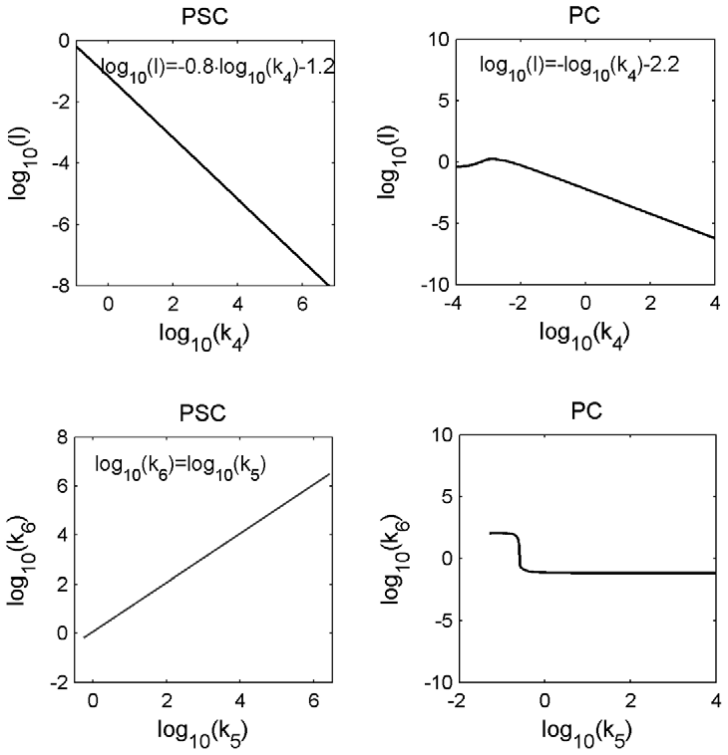
Correlations between model parameters after calculation of the PLEs. The parameter for phosphorylation (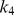) is inversely correlated to the total receptor concentration (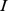) for PSC and PC as presented in the upper row. The lower row, left column shows the correlation between the parameters nuclear dephosphorylation (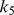) and nuclear import of STAT1Dc (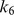) and for PSC. A respective correlation equation is inserted in each subfigure. The lower row, right column shows for PC: One finds 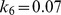 min^−1^ which is located inside the CI for 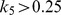 min^−1^. However, one finds 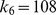 min^−1^ which is located outside the upper boundary of its CI for 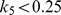 min^−1^.

**Figure 8:**
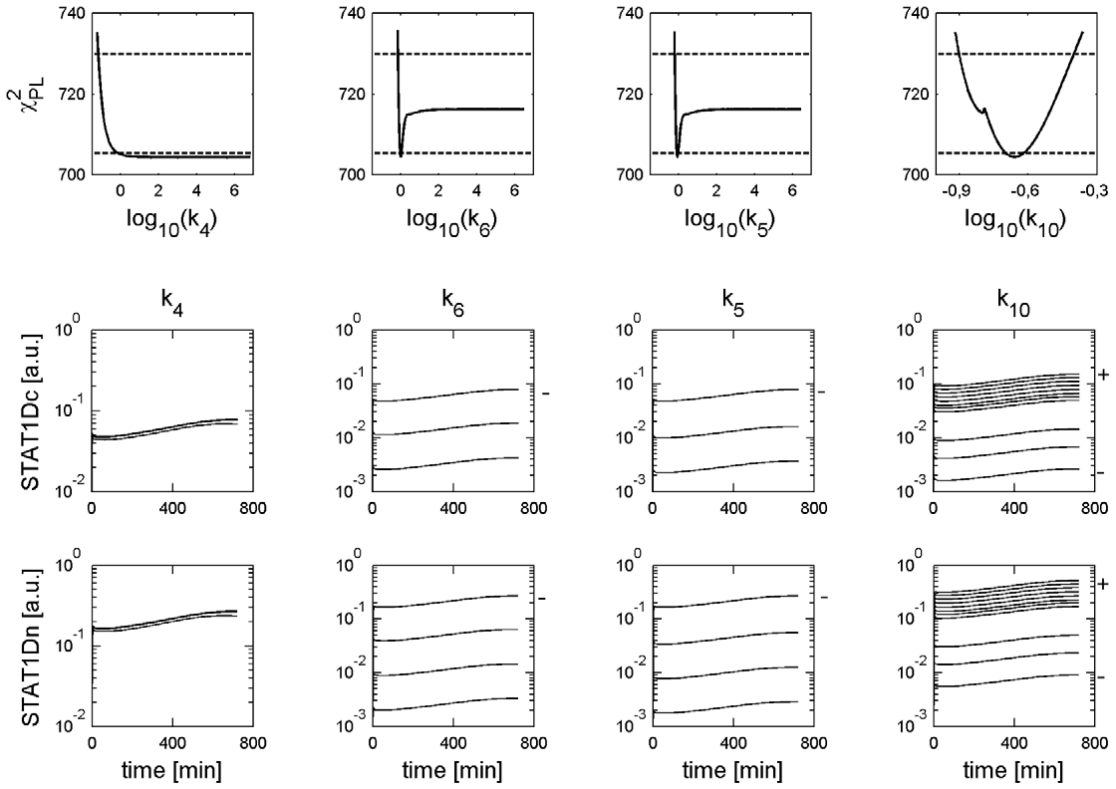
Trajectories for parameter sets along profile likelihoods show nuclear accumulation of phosphorylated STAT1 in PSC. The upper row shows the PLEs of the parameters 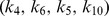 from Figure 3. The lower rows show trajectories generated from 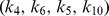 parameter sets within the PLE confidence intervals of the variables cytoplasmic and nuclear phosphorylated STAT1 (STAT1Dc,STAT1Dn). Stimulation was done with IFNγ = 100 ng/ml. For higher values of 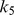 and 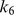 the plateau of the PLE trajectories further decrease, such that for 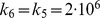 min^−1^ the plateau is located at 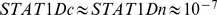 a.u. For further details see captions of Figure 6.

**Figure 9:**
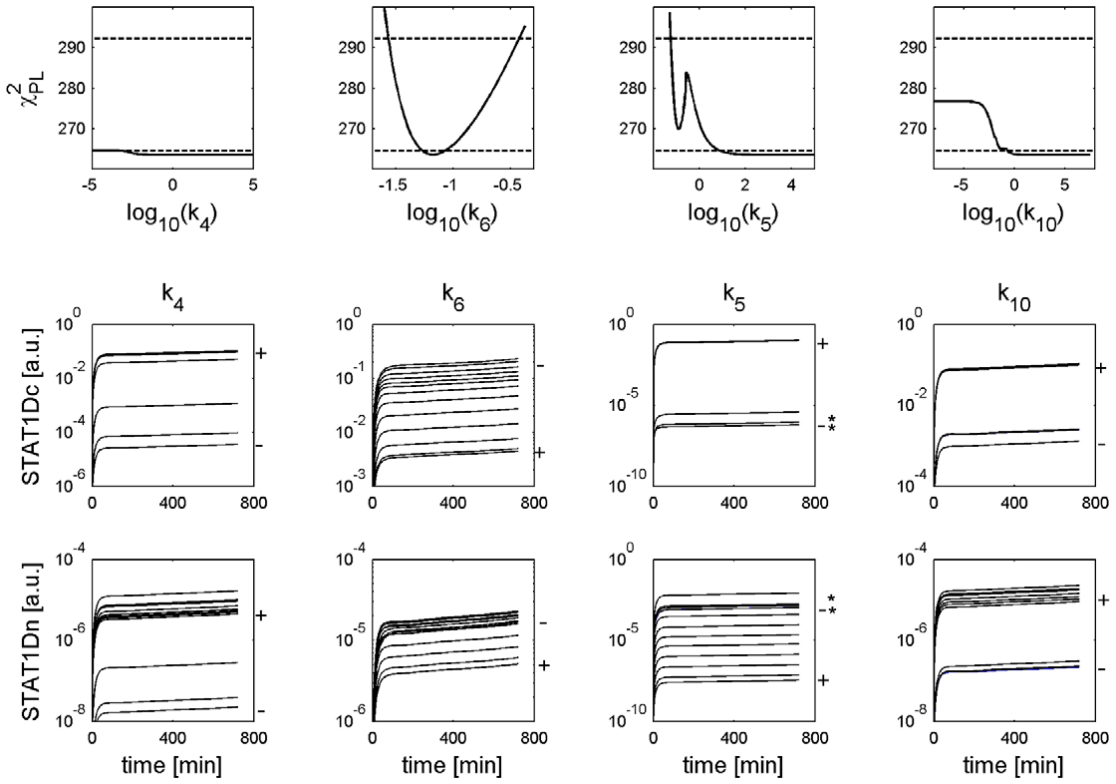
Trajectories for parameter sets along profile likelihoods show missing nuclear accumulation of phosphorylated STAT1 in PC. The upper row shows the PLEs of the parameters 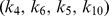 from Figure 4. The lower rows show trajectories generated from 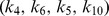 parameter sets within the PLE confidence intervals of the variables cytoplasmic and nuclear phosphorylated STAT1 (STAT1Dc, STAT1Dn). Stimulation was done with IFNγ = 100 ng/ml. A “*” indicates trajectories for 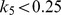 min^−1^. In this region one finds 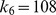 min^−1^ (see Figure 7), which is located outside the upper boundary of its CI. For further details see captions of Figure 6.

- Rapid initial increase of phosphorylated STAT1 time series in PSC in contrast to PC as presented in Figure 2 - upper row.
  - The estimated value of the parameter 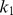 (IFNγ receptor activation) is larger in PSC than in PC and the CIs do not overlap (Figure 6 - upper row). Simulations for parameter sets within the CI of 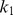 show that PSC trajectories for activated receptor (IIr) and phosphorylated STAT1 (STAT1D) rapidly increase in contrast to the respective trajectories for PC, see Figure 6 - second and third row.
  - The parameter 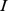 (total receptor concentration) is inverse proportional to 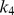 (STAT1 phosphorylation) for PSC and PC, as presented in the upper row of Figure 7. The correlation equation presented in the respective subfigures show that the product 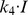 is larger for PSC than for PC contributing to a more rapid phosphorylation of STAT1 in PSC.
- Nuclear accumulation of STAT1 in PSC but not in PC, see Figure 2 - lower row.
  - The negative correlation between 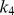 and 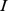 contributes to the small variability of the 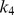 trajectories for cytoplasmic and nuclear phosphorylated STAT1 (STAT1Dc, STAT1Dn) of the PSC model in contrast to the variability of the PLE trajectories of other parameters, see Figure 8. The parameters 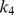 and 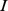 are only negatively correlated for 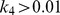 min^−1^a.u.^−1^ in the PC model. This leads to a broader variability of the 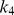 trajectories resulting from the PC model, see Figure 9.
  - The parameter 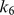 describing nuclear import of phosphorylated STAT1 is correlated with 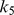 describing nuclear dephosphorylation in the PSC model, see Figure 7 lower row. The ratio of 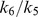 is nearly equal to one. This leads to a rapid nuclear accumulation of phosphorylated STAT1 and to a temporal constant ratio of nuclear versus cytoplasmic phosphorylated STAT1 (Figure S1 in Text S1, subfigure “STAT1D immunofluorescence”). Trajectories, generated from the PLEs of parameters involved in STAT1 activation, deactivation and transport 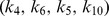, show that IFNγ induced STAT1 phosphorylation is followed by a 3.5-fold increased nuclear accumulation of phosphorylated STAT1, relative to the cytoplasm (Figure 8 - lower rows). The parameter 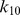 describing nuclear export is identifiable and the upper boundary of its CI is smaller than the lower boundary of the 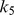-CI (Figure 8 - upper row). This leads to an accumulation of unphosphorylated STAT1 in the nucleus which is counterbalanced by enhanced STAT1 expression at times greater than 200 min. These reactions together explain the temporal increase with an initial overshoot of the ratio nuclear versus cytoplasmic STAT1 for PSC as shown in Figure 2.
  - The ratio of nuclear versus cytoplasmic STAT1 for PC does not show a nuclear accumulation of STAT1, as depicted in Figure 2. The parameter 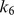 is identifiable, the best fit value is lower than the best fit value for PSC and the CIs do not overlap, see the respective PLEs in the upper row of Figures 8 and 9. Trajectories generated from the PLEs of parameters involved in STAT1 activation, deactivation and transport 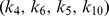 show that STAT1 gets phosphorylated but it mainly remains in the cytoplasm (lower rows in Figure 9). Few trajectories of 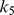 in Figure 9 which contradict this conclusion base on values of 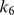 located outside the upper boundary of its CI. Figure 7 depicts: For 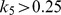 min^−1^ one finds 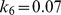 min^−1^, which is inside the boundaries of the CI, but for 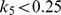 min^−1^ one finds 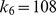 min^−1^ which is located outside the upper boundary of its CI.

Summarizing our results, we successfully identified reactions that could explain the differences of STAT1 signalling in pancreatic stellate and cancer cells: A larger value of the reaction constant for receptor activation (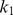) leads to a rapid increase of STAT1D in PSC and to a slower increase of STAT1D in PC, see Figures 2 and 6. A larger value of the reaction constant for nuclear import of phosphorylated STAT1 (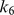) leads to a nuclear accumulation of STAT1 in PSC and to a lack of nuclear accumulation of STAT1 in PC, as illustrated in Figures 2, 8, and 9.

The parameter identifiability analysis revealed additional insights into consequences of parameter uncertainties: The negative correlation between the parameters 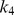 and 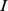 in the PSC model contributes to the small variability of the 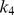-trajectories of STAT1Dc and STAT1Dn. The parameter describing nuclear export (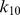) is identifiable in PSC and the upper boundary of its CI is smaller than the lower boundary of the 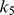-CI. An open question is whether the resulting nuclear accumulation of STAT1Un supports IFNγ action too.

### Sensitivity analysis

**Figure 10:**
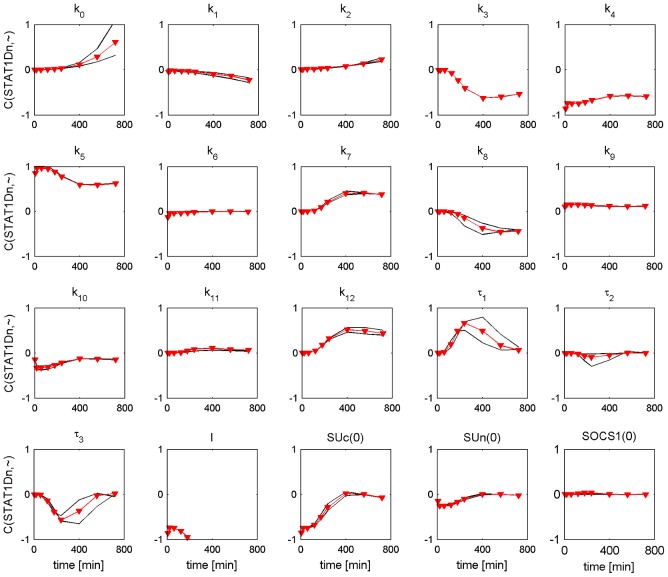
Time dependent metabolic control coefficients for nuclear phosphorylated STAT1 of PSC model. Stimulation with IFNγ = 100 ng/ml. The observation time is given on the x-axis of each subfigure. The metabolic control coefficient is given on the y-axis. The symbol “∼” in the y-axis label is a placeholder for the respective parameter or initial condition name. Each parameter or initial condition is independently perturbed by −1%. The metabolic control coefficients for the perturbed default parameter set (See Tables S1, S2, S3 in Text S1) are shown as red triangles. The black lines show the lower and upper CI boundaries of the MCCs. For few parameters one or both black lines are behind the red line.

**Figure 11:**
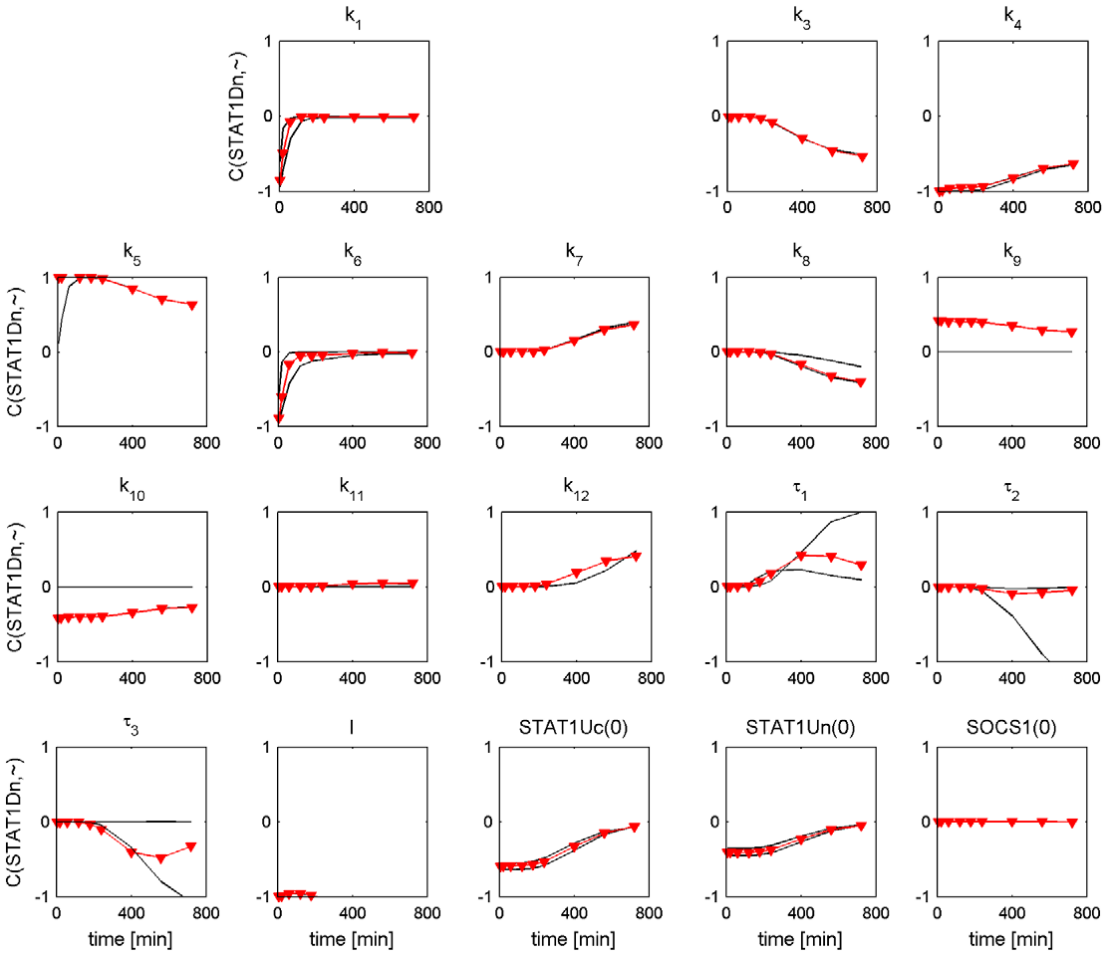
Time dependent metabolic control coefficients for nuclear phosphorylated STAT1 of PC model. Stimulation with IFNγ = 100 ng/ml. The observation time is given on the x-axis of each subfigure. The metabolic control coefficient is given on the y-axis. The symbol “∼” in the y-axis label is a placeholder for the respective parameter or initial condition name. Each parameter or initial condition is independently perturbed by −1%. The metabolic control coefficients for the perturbed default parameter set (See Tables S1, S2, S3 in Text S1) are shown as red triangles.The black lines show the lower and upper CI boundaries of the MCCs. For few parameters one or both black lines are behind the red line.

In this section we investigated the influence of parameter perturbations on the trajectories of nuclear phosphorylated STAT1, which we consider as the output of the pathway. We calculated the metabolic control coefficient for different time points according to Eq. (3) in order to find out which perturbed parameters most sensitively influence the temporal profile of nuclear phosphorylated STAT1 in the PSC and PC model. The default parameter values are presented in Tables S1, S2, S3 in Text S1. The calculation was performed for 20 parameter value sets covering the CI of each default parameter value. The results for the time-dependent metabolic control coefficients are shown in Figures 10 and 11. The coefficients with the largest deviation from the default coefficient form the CI boundaries of the default coefficient. Interestingly, the temporal profiles of the metabolic control coefficients for the different parameter values are similar between the PSC parameter sets (Figure 10) and the PC parameter sets (Figure 11). The CI boundaries are located close to the default coefficients in the PSC model. This holds also for most parameters in the PC model.

The results show that nuclear phosphorylated STAT1 sensitively depends on perturbed parameters describing phosphorylation (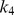), dephosphorylation (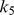) and total receptor concentration (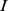) over the whole time interval but it is robust against perturbations of phosphorylated STAT1 nuclear import 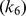. While 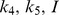 are nonidentifiable parameters in both models the parameter 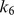 is nonidentifiable in the PSC model but identifiable in the PC model.

For the control coefficients of the PC model 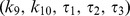 the CI boundaries show some deviations from the default control coefficients leading to a change of sensitivity from medium to robust. The parameter 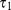 is identifiable, while the other four parameters are nonidentifiable.

Summarizing, our results show that there is no clear relation between parameter identifiability and sensitivity. Note that parameter identifiability can be improved by additional experiments, while sensitivity only depends on the model properties. An explanation for the sensitive response of the nonidentifiable parameters 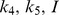 in the PSC and PC model could be: Perturbing a parameter value can lead to a very sensitive response of a model variable. However, parameter identifiability analysis proceeds by fitting again the other parameter values. This could lead to very similar or even identical 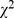 values.

## Discussion

### Parameter identifiability analysis

Our results of the parameter identifiability analysis led to a successful identification of model parameters and parameter relations, explaining differences between the experimental time series of STAT1 phosphorylation and STAT1 nuclear accumulation for pancreatic stellate cells and pancreatic cancer cells.

We verified whether the normalized frequency distribution 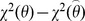 follows a 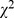 distribution to test whether the initially chosen confidence level is consistent with the calculated confidence intervals for the parameter values according to [20], [21]. We obtained the best agreement for an “effective number” of degrees of freedom of the 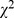 distribution, see Figure 5. Based on the reduced number of degrees of freedom we obtained a higher confidence level for the calculated confidence intervals of the parameter value estimates. The calculation of confidence intervals for the parameter values of the best fit allowed a comparison between the parameter variability and the variability in the model trajectories, as presented in Figures 6, 8 and 9. Trajectories for parameter sets within the confidence interval show:

- The best fit value of the parameter describing receptor activation (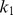) is larger for the PSC model than for the PC model and the confidence intervals do not overlap. This leads to a faster increase of phosphorylated STAT1 in PSC, compared to PC.
- The best fit value of the parameter describing nuclear translocation of phosphorylated STAT1 (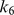) is larger for the PSC model than for the PC model and the confidence intervals do not overlap. This leads to a high nuclear accumulation of STAT1 in PSC in contrast to PC.

We successfully answered the first question raised in the introduction, asking for what causes the differences between the temporal profiles in pancreatic stellate cells and cancer cells. Our analysis showed that valuable information for model comparison can also be obtained from nonidentifiable parameters. Correlated parameters and parameters with one finite confidence interval helped to explain differences between IFNγ-induced STAT1 signalling in stellate and cancer cells. This suggests that one should extend those parameter regions for which a profile likelihood estimate was calculated, either until a confidence interval boundary has been reached or until a further calculation is limited by numerical precision.

We are, however, also aware of the limitations that are due to parameter nonidentifiabilities in our studied examples. Reducing practical nonidentifiability, by decreasing the experimental noise, would improve the differentiation between the parameter sets (including CI) that fit the PSC time series and the parameter sets (including CI) that fit the PC time series. A model variable which is a nonobservable can be influenced by changing a parameter value even within its CI. This includes the amount of phosphorylated STAT1 or to answer the question whether there is more nuclear phosphorylated STAT1 in one of the two cell types. Nevertheless, we positively exploited information from nonidentifiability in our work: The knowledge of one finite confidence interval boundary of a nonidentifiable parameter was sufficient to draw conclusions about reactions which differ between the IFNγ induced STAT1 signalling pathway in pancreatic stellate cells and pancreatic cancer cells.

### Sensitivity analysis

We used the results of the parameter identifiability analysis to choose parameter sets within the confidence intervals for the sensitivity analysis. The profiles of the time-dependent metabolic control coefficients are very similar for the PSC parameter sets and the PC parameter sets as demonstrated in Figures 10 and 11. Though only few parameters turned out to be identifiable, our study demonstrated that the results from sensitivity analysis are largely robust for different parameter sets within the CIs and moreover the results from sensitivity analysis are largely robust when comparing the two cell types. Our findings successfully answer the second question raised in the introduction, asking for the consequences of differences between the model simulations in pancreatic stellate cells and cancer cells for therapeutic target prediction. The first outcome is also in line with Chen et al., who found that for the ErbB1-4 receptors activated MAPK and P13K/Akt pathway in cancer cell types several parameter sets with similar good fits do not affect sensitivity [25].

Our results show that the time dependent metabolic control coefficients can be used for experimental therapeutic studies in cell culture. A similar question was asked by Raia et al., who predicted that the inhibition of STAT5 phosphorylation in IL13 induced STAT5 pathway reduces proliferation in two lymphoma cell types [12]. However, in our investigation we need to enhance STAT1 phosphorylation, in order to ensure a stronger reduction of proliferation in both cell types [4], [5]. An interesting complement or alternative to this approach is targeting intracellular components because a higher IFNγ dose could lead to unwanted side effects. A reduction of proliferation by maximization of nuclear phosphorylated STAT1 could be achieved by inhibiting nuclear dephosphorylation of STAT1 (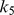), SOCS1 transcription (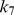), and the feedback regulation (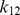) as depicted in Figures 10 and 11. Inhibition of dephosphorylation shows an effect over the whole observation time while the other two processes are sensitive only after a time delay. Simulations with 50% inhibition of 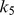 are in agreement with the results for the metabolic control coefficients with 1% inhibition, see Figure S3 in Text S1.

### Nuclear accumulation of STAT1- bringing the pieces together

We used mathematical modeling, simulations and parameter identifiability analysis to explain experimentally observed differences of IFNγ-induced STAT1 signalling in pancreatic stellate cells and cancer cells. Parameter identifiability analysis revealed that it is a larger value of the reaction constant for nuclear import of phosphorylated STAT1 (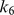) that leads to a nuclear accumulation of STAT1 in PSC but not in PC.

**Figure 12:**
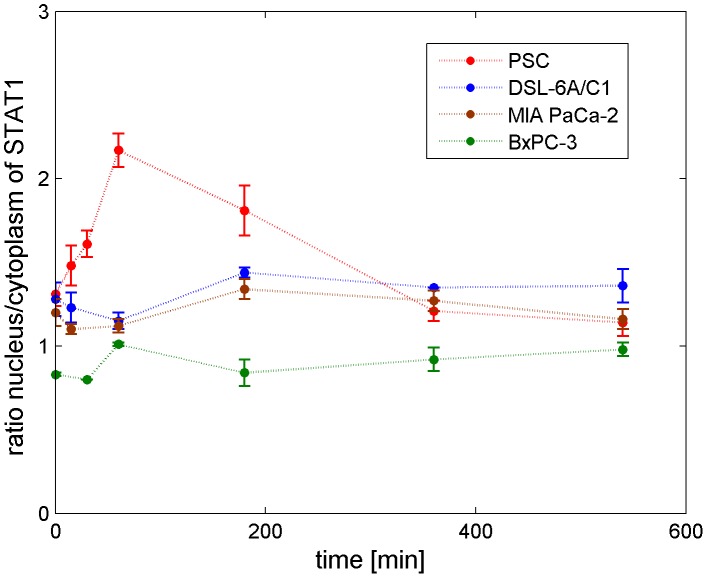
Nuclear accumulation of STAT1 in primary PSC and PC cells. Isolated primary PSC from rat pancreas and PC cell lines of rat (DSL-6A/C1) and human (BxPC-3, MIA PaCa-2) origin were stimulated with 100 ng/ml species-specific IFNγ for the indicated times. STAT1 nuclear translocation was detected by immunofluorescence analysis. Data obtained by confocal microscopy were processed by calculating the ratio of nuclear versus cytoplasmic STAT1 concentration. Measured data are presented as circles with error bars, mean (n≥10) ± SEM.

Subsequently, we expanded our experimental studies to primary rat PSC and different human PC cell lines, and performed additional time course experiments. The cells were stimulated with 100 ng/ml IFNγ and STAT1 translocation was observed for nine hours. We could experimentally confirm the nuclear accumulation of STAT1 after IFNγ treatment also for primary PSC, as presented in Figure 12. In contrast, an extended time series of the PC cell line DSL-6A/C1 showed no nuclear accumulation of STAT1 in response to IFNγ. This result agrees with the shorter time series in Figure 2. A nuclear accumulation of STAT1 was also not observed in the human PC cell lines BxPC-3 and MIA PaCa-2 (Figure 12). Our results therefore allow the conclusion that the differences in IFNγ induced nuclear accumulation of STAT1 are not restricted to the two originally used cell lines but reflect a general difference between pancreatic stellate and cancer cells. In two previous studies, we could show that nuclear accumulation of STAT1 is directly correlated with the antiproliferative effect of IFNγ. Accordingly, pancreatic stellate cells were found to be more sensitive to IFNγ-mediated growth inhibition than pancreatic cancer cells [4], [5].

The nuclear accumulation of STAT1 is induced by STAT1 phosphorylation. The parameter identifiability analysis of the ODE models led to the result that the nuclear import of STAT1D (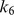) is a parameter with a distinct value in the ODE models of PSC and PC, see Figures 8 and 9, upper row. However, the results of our sensitivity analysis reveal that the trajectories of nuclear phosphorylated STAT1, which we consider as output of the pathway, are robust against perturbations of the parameter 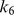, see Figures 10 and 11. Remarkable, the nuclear import of STAT1D cannot be considered as a target for experimental therapeutic studies in cell culture.

An alternative parameter, which can influence the nuclear accumulation of STAT1, is the nuclear dephosphorylation (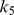). This parameter is nonidentifiable in the PSC and PC model and the finite lower CIs do not provide information which parameter value is larger. However, results of the sensitivity analysis reveal that nuclear dephosphorylation of STAT1 (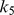) is the most sensitive parameter for both cell types independent of the chosen parameter set within the CI. The inhibition of 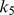 leads to a nuclear accumulation of STAT1 over the whole measured period. The parameter describing nuclear dephosphorylation of STAT1D (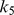) can thus be considered as a target for experimental *in vitro* therapeutic studies.

### Conclusions

We demonstrated that results from parameter identifiability analysis can be exploited to designate reactions of a signalling pathway which differ between two cell types. In particular, even information from nonidentifiable parameters contributed to our findings. Interestingly, the sensitivity of model variables is robust against nonidentifiability and moreover it is robust against the different parameterizations for the two cell types. This enabled us to predict targets that can be successfully hit in both cell types despite quantitative and temporal differences in the activation profile of the signaling cascade. At the current stage, our results and conclusions are restricted to *in vitro* models. In the long run, they may also contribute to the development of drugs that display optimized simultaneous effects on different types of cells, e.g. in the context of cancer.

## Supporting Information

Text S1 **We present the mathematical model, model parameters, supplementary figures and a list of abbreviations.**
(PDF)
