# Supplementary material for: Parameter Identifiability and Sensitivity Analysis Predict Targets for Enhancement of STAT1 Activity in Pancreatic Cancer and Stellate Cells
Source: PLoS Comput Biol. 2012 Dec 20;8(12):e1002815. doi: 10.1371/journal.pcbi.1002815 (PMC3527226; doi:10.1371/journal.pcbi.1002815)
Supplement: Text S1 — We present the mathematical model, model parameters, supplementary figures and a list of abbreviations. (PDF) [file pcbi.1002815.s001.pdf]

Supporting Information
Text S1

Parameter Identifiability and Sensitivity Analysis Predict Targets for Enhancement of STAT1 Activity in Pancreatic Cancer and Stellate Cells

Katja Rateitschak[1,*], Felix Winter[1], Falko Lange[1,2], Robert Jaster[2], Olaf Wolkenhauer[1,3]

[1]Department of Systems Biology and Bioinformatics, University of Rostock, 18051 Rostock, Germany

[2]Department of Medicine II, Division of Gastroenterology, Medical Faculty, University of Rostock, 18057 Rostock, Germany

[3]Stellenbosch Institute for Advanced Study (STIAS), Wallenberg Research Center at Stellenbosch University, Marais Street, Stellenbosch 7600, South Africa

*Corresponding author: katja.rateitschak@uni-rostock.de

Content:

1. Mathematical model

2. Tables with parameter values

3. Supplementary Figures

4. List of abbreviations

1. Mathematical model

Ordinary differential equation model for PSC and PC

$$\frac{d}{dt} IFN\gamma = -k_0 \cdot IFN\gamma - k_1 \cdot IFN\gamma \cdot (I - IIr) + k_2 \cdot IIr$$
$$\frac{d}{dt} IIr = k_1 \cdot IFN\gamma \cdot (I - IIr) - k_2 \cdot IIr$$
$$\frac{d}{dt} STAT1Uc = k_3 \cdot \int_0^\infty \Gamma_{q_1}^4(\tau) \cdot IIr(t-\tau)d\tau - k_4 \cdot IIr \cdot STAT1Uc/(1 + k_{12} \cdot \int_0^\infty \Gamma_{q_3}^4(\tau) SOCS1(t-\tau)d\tau) - k_9 \cdot STAT1Uc + \frac{k_{10}}{k_v} \cdot STAT1Un$$
$$\frac{d}{dt} STAT1Dc = k_4 \cdot IIr \cdot STAT1Uc/(1 + k_{12} \cdot \int_0^\infty \Gamma_{q_3}^4(\tau) SOCS1(t-\tau)d\tau) - k_6 \cdot STAT1Dc$$
$$\frac{d}{dt} STAT1Dn = k_v \cdot k_6 \cdot STAT1Dc - k_5 \cdot STAT1Dn$$
$$\frac{d}{dt} STAT1Un = k_v \cdot k_9 \cdot STAT1Uc - k_{10} \cdot STAT1Un + k_5 \cdot STAT1Dn$$
$$\frac{d}{dt} SOCS1 = k_{11} + k_7 \cdot \int_0^\infty \Gamma_{q_2}^4(\tau) \cdot STAT1Dn(t-\tau)d\tau - k_8 \cdot SOCS1$$

The delayed processes are described by a distributed time delay with mean delay time $\bar{\tau}_i$. Kernel of the Gamma function is described as:

$$\Gamma_{q_i}^p(\tau) = \frac{q_i^{\,p}}{(p-1)!} \tau^{p-1} \cdot e^{-q_i \tau} \quad p = 4 \quad q_i = \frac{p}{\bar{\tau}_i}$$

The shape is determined by the parameters p and $\bar{\tau}_i$. The parameters k_i are reaction constants, I is the total receptor concentration and k_v is the ratio between cytoplasmic and nuclear size. It has the value 3 for PSC and 1 for PC. The variables have arbitrary units of concentration.

Relations between observables and model variables for the PSC model

$$STAT1 = 3/4 \cdot (STAT1Dc + STAT1Uc) + 1/4 \cdot (STAT1Dn + STAT1Un)$$
$$STAT1D = (3 \cdot STAT1Dc/4 + STAT1Dn/4) \cdot WB_{STAT1D}$$
$$SOCS1 = SOCS1 \cdot PCR_{SOCS1}$$
$$RSNC = \frac{(STAT1Dn + STAT1Un)}{(STAT1Dc + STAT1Uc)}$$
$$RSPNC = \frac{STAT1Dn}{STAT1Dc}$$

Relations between observables and model variables for the PC model

$$STAT1 = (STAT1Dc + STAT1Uc + STAT1Dn + STAT1Un)/2$$
$$STAT1D = (STAT1Dc + STAT1Dn)/2 \cdot WB_{STAT1D}$$
$$SOCS1 = SOCS1$$
$$RSNC = \frac{(STAT1Dn + STAT1Un)}{(STAT1Dc + STAT1Uc)}$$
$$STAT1c = (STAT1Dc + STAT1Uc) \cdot WB_{STAT1c}$$
$$STAT1n = (STAT1Dn + STAT1Un) \cdot WB_{STAT1n}$$

$$STAT1Dc = STAT1Dc \cdot WB_{STAT1Dc}$$
$$STAT1Dn = STAT1Dn \cdot WB_{STAT1Dn}$$

The numbers in some equations are based on a three times larger cytoplasmic size than nuclear size in PSC and an equal size of both compartments in PC. The parameters WB_{STAT1x} are Western blot and PCR_{SOCS1} are real time PCR scaling factors. The scaling factors PCR_{SOCS1} (for PSC, IFNγ=1 ng/ml and PC) and WB_{STAT1} are redundant parameters which are fixed to the value 1.

2. Tables with parameter values

Table S1

**Global parameters**			
	**PSC**	**PC**	
Parameter	Value	Value	Unit
k_0	0.004	0	min^{-1}
k_1	0.02	0.0009	$\text{min}^{-1}\,\text{a.u.}^{-1}$
k_2	0.04	0	min^{-1}
k_3	10	0.096	min^{-1}
k_4	50	0.1	$\text{min}^{-1}\,\text{a.u.}^{-1}$
k_5	0.84	298	min^{-1}
k_6	0.96	0.067	min^{-1}
k_7	0.11	4180	min^{-1}
k_8	0.01	0.06	min^{-1}
k_9	0.06	8.9	min^{-1}
k_{10}	0.22	12.3	min^{-1}
k_{11}	0.005	0.009	$\text{min}^{-1}\,\text{a.u.}$
k_{12}	1.2	0.75	min^{-1}
$\bar{\tau}_1$	201	277	min
$\bar{\tau}_2$	37	79	min
$\bar{\tau}_3$	228	452	min
I	0.001	0.06	a.u.
$STAT1Uc(0)$	0.91	0.95	a.u.
$STAT1Un(0)$	0.91	0.66	a.u.
$SOCS1(0)$	0.21	0.11	a.u.

Note that for the PC model, the parameters for IFNγ degradation k_0 and IFNγ receptor complex dissociation k_2 have been estimated to zero with precision 10^{-8}.

Table S2

Local parameters			
		**PSC**	**PC**
IFNγ (ng/ml)	Parameter	Value	Value
100	WB_{STAT1D}	12	34
	$WB_{STAT1Dc}$		19
	$WB_{STAT1Dn}$		91678
	WB_{STAT1c}		0.75
	WB_{STAT1n}		1.22
	PCR_{SOCS1}	0.57	
10	WB_{STAT1D}		44
1	WB_{STAT1D}	26	

Table S3

Not optimized parameters			
	**PSC**	**PC**	
Parameter	Value	Value	Unit
IIr	0	0	a.u.
$STAT1Dc(0)$	0	0	a.u.
$STAT1Dn(0)$	0	0	a.u.

3. Supplementary Figures

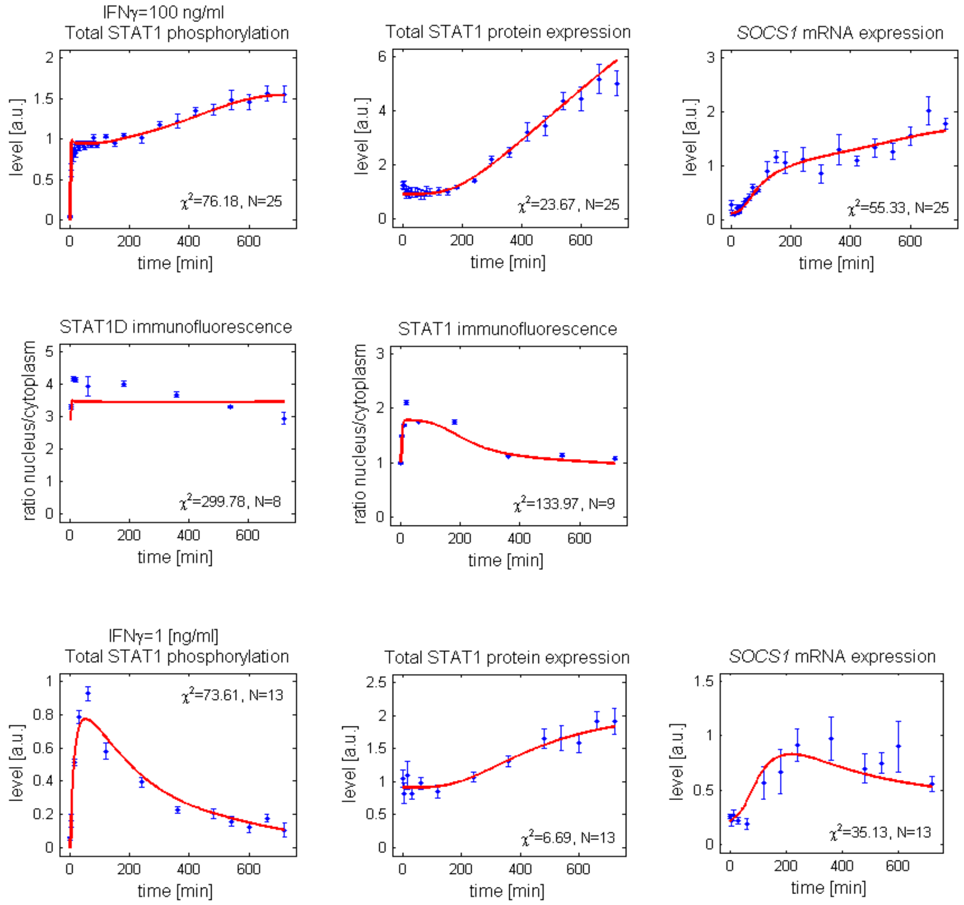

Fig. S1. IFNγ-induced STAT1 signal transduction in PSC: Comparison between experimental time series and model simulations. Upper rows: IFNγ = 100 ng/ml, Lower row: IFNγ = 1 ng/ml. The observation time is given on the x-axis of each subfigure. Experimentally determined expression levels of phospho-STAT1, total STAT1 protein and *SOCS1* mRNA are given in arbitrary units (a.u.). Immunofluorescence analysis by confocal microscopy was processed by calculating the ratio of nuclear versus cytoplasmic STAT1 concentration and phosphorylated STAT1 concentration respectively. The immunofluorescence signal of phosphorylated STAT1 was not quantifiable at t=0 min. Measured data are presented as blue circles with error bars. The simulated time courses resulting from the mathematical model with optimized parameter values for STAT1, nuclear translocation of STAT1, phosphorylated STAT1 and *SOCS1* mRNA are presented by red solid lines. Experimental time series are replotted from [6], except the new time series for STAT1D immunofluorescence.

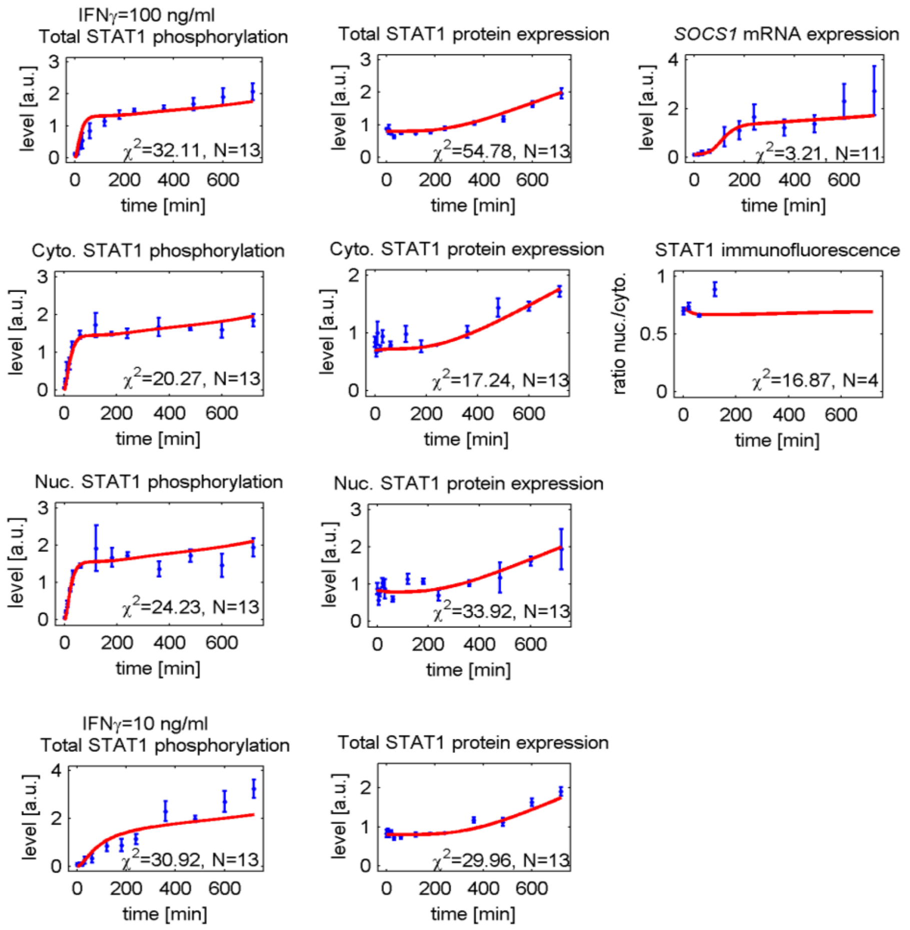

Fig. S2. IFNγ-induced STAT1 signal transduction in PC: Comparison between experimental time series and model simulations. Upper rows: IFNγ = 100 ng/ml, Lower row: IFNγ = 10 ng/ml. Further explanations are the same as in captions of Fig. S1. Different profiles of STAT1D for smaller concentrations of IFNγ between the cell types are reflected in the results of the parameter estimation: IFNγ degradation (k_0) and receptor deactivation (k_2) are estimated to zero for PC, see Table S1 in Text S1. At 1 ng/ml IFNγ did not activate STAT1 at all in PC. Comparing the subfigures for IFNγ = 100 ng/ml shows a faster initial slope in the experimental time series and model simulations for the cytoplasmic and nuclear extracts STAT1Dc and STAT1Dn in contrast to the slower initial slope of the experimental time series for total STAT1D. This caused the less good quality of the fit for total STAT1D. By repeating the fitting we tried to constrain parameters to capture better the fit for STAT1D eventually on cost of the fits for STATDc and STAT1Dn. Unfortunately this effort did not changed the fit. Experimental time series are replotted from [5].

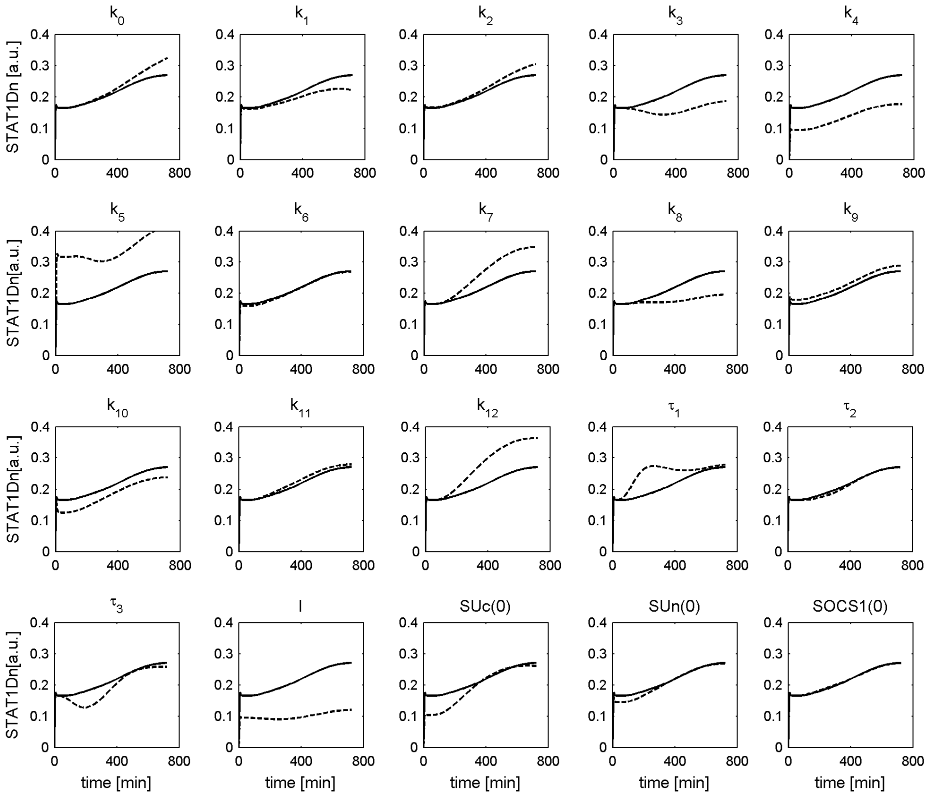

Fig. S3. Sensitivity analysis, perturbed trajectories for PSC. Stimulation with IFNγ = 100 ng/ml. Observation time is given on the x-axis of each subfigure. The concentration of nuclear phosphorylated STAT1 is given on the y-axis. Solid line: unperturbed trajectory. Dashed line: Trajectory resulting from parameter perturbation of –50 %.

4. List of abbreviations

a.u.: arbitrary unit; CI: confidence interval; df: degree of freedom; I: total receptor concentration; IFN: interferon; IIr: active IFNγ receptor; Ir: inactive IFNγ receptor; PC: pancreatic cancer; PSC: pancreatic stellate cell; PLE: profile likelihood estimate; RSNC: ratio of nuclear versus cytoplasmic concentration of STAT1; RSPNC: ratio of nuclear versus cytoplasmic concentration of phosphorylated STAT1; SOCS: suppressor of cytokine signalling; STAT: signal transducer and activator of transcription; STAT1c: STAT1 in the cytoplasm; STAT1n: STAT1 in the nucleus; STAT1D: phosphorylated STAT1 dimer; STAT1Dc: phosphorylated STAT1 dimer in the cytoplasm; STAT1Dn: phosphorylated STAT1 dimer in the nucleus; STAT1U: unphosphorylated STAT1; STAT1Uc: unphosphorylated STAT1 in the cytoplasm; STAT1Un: unphosphorylated STAT1 in the nucleus
